# Novel therapeutic perspectives in Noonan syndrome and RASopathies

**DOI:** 10.1007/s00431-023-05263-y

**Published:** 2023-10-21

**Authors:** Céline Saint-Laurent, Laurène Mazeyrie, Armelle Yart, Thomas Edouard

**Affiliations:** 1 RESTORE Research Center, Université de Toulouse, Institut National de La Santé Et de La Recherche Médicale 1301, Centre National de La Recherche Scientifique 5070 Toulouse, France; 2 Endocrine, Bone Diseases, and Genetics Unit, Reference Center for Endocrine Diseases of Growth and Development, FIRENDO Network, Children’s Hospital, Toulouse University Hospital, 330 Avenue de Grande-Bretagne TSA 70034, 31059 Toulouse Cedex 9, France

**Keywords:** Noonan syndrome, RASopathies, RAS/MAPK signalling pathway, MEK inhibitors, C-type natriuretic peptide analogues, Statins

## Abstract

Noonan syndrome belongs to the family of RASopathies, a group of multiple congenital anomaly disorders caused by pathogenic variants in genes encoding components or regulators of the RAS/mitogen-activated protein kinase (MAPK) signalling pathway. Collectively, all these pathogenic variants lead to increased RAS/MAPK activation. The better understanding of the molecular mechanisms underlying the different manifestations of NS and RASopathies has led to the identification of molecular targets for specific pharmacological interventions. Many specific agents (e.g. SHP2 and MEK inhibitors) have already been developed for the treatment of RAS/MAPK-driven malignancies. In addition, other molecules with the property of modulating RAS/MAPK activation are indicated in non-malignant diseases (e.g. C-type natriuretic peptide analogues in achondroplasia or statins in hypercholesterolemia).

*Conclusion*: Drug repositioning of these molecules represents a challenging approach to treat or prevent medical complications associated with RASopathies.

**Table:** 

**What is Known:** *• Noonan syndrome and related disorders are caused by pathogenic variants in genes encoding components or regulators of the RAS/mitogen-activated protein kinase (MAPK) signalling pathway, resulting in increased activation of this pathway.* *• This group of disorders is now known as RASopathies and represents one of the largest groups of multiple congenital anomaly diseases known.*	
**What is New:** *• The identification of pathophysiological mechanisms provides new insights into the development of specific therapeutic strategies, in particular treatment aimed at reducing RAS/MAPK hyperactivation.* *• Drug repositioning of specific agents already developed for the treatment of malignant (e.g. SHP2 and MEK inhibitors) or non-malignant diseases (e.g. C-type natriuretic peptide analogues in achondroplasia or statins in hypercholesterolaemia) represents a challenging approach to the treatment of RASopathies.*

**What is Known:**

*• Noonan syndrome and related disorders are caused by pathogenic variants in genes encoding components or regulators of the RAS/mitogen-activated protein kinase (MAPK) signalling pathway, resulting in increased activation of this pathway.*

*• This group of disorders is now known as RASopathies and represents one of the largest groups of multiple congenital anomaly diseases known.*

**What is New:**

*• The identification of pathophysiological mechanisms provides new insights into the development of specific therapeutic strategies, in particular treatment aimed at reducing RAS/MAPK hyperactivation.*

*• Drug repositioning of specific agents already developed for the treatment of malignant (e.g. SHP2 and MEK inhibitors) or non-malignant diseases (e.g. C-type natriuretic peptide analogues in achondroplasia or statins in hypercholesterolaemia) represents a challenging approach to the treatment of RASopathies.*

## Introduction: Noonan syndrome and RASopathies

Noonan syndrome (NS; MIM # 163,950) is a relatively common developmental disorder (estimated incidence of 1/2000 live births) that can affect multiple organ systems with variable severity [1]. The diagnosis of NS is usually based on clinical features, including distinctive facial features, congenital heart defects (i.e. pulmonary valve stenosis and hypertrophic cardiomyopathy), short stature, skeletal abnormalities (i.e. pectus, scoliosis), variable cognitive impairment, cryptorchidism in males, and lymphatic dysplasia. Other features include an increased risk of developing malignancies (such as juvenile myelomonocytic leukaemia, neuroblastoma, low-grade glioma, giant cell lesions, and rhabdomyosarcoma) [2] and metabolic dysfunction [3]. This syndrome is characterised by a high degree of phenotypic variability underlined by genetic heterogeneity. Indeed, germline pathogenic variants have been identified in at least 12 different genes in patients with NS features [4]. Approximately half of NS patients carry a germline missense mutation in the *PTPN11* gene, which encodes the tyrosine phosphatase SHP2, along with less common mutations in *SOS1* (10%), *RIT1* (10%), *RAF1* (5%), *KRAS* (5%), and other rarer genes (*BRAF*, *LZTR1*, *SOS2*, *NRAS*, *RRAS*, *RRAS2*, and *MRAS* genes). Genetic screening remains negative in about 10–20% of patients with NS. Interestingly, all of these genes encode components or regulators of the RAS/mitogen-activated protein kinase (MAPK) signalling pathway, which plays a key role in important cellular processes such as proliferation, survival, differentiation, and metabolism. Collectively, all these pathogenic variants lead to increased RAS/MAPK activation [5]. Constitutional dysregulation of the RAS/MAPK pathway also causes phenotypically overlapping disorders with NS, including NS with multiple lentigines (NSML), Noonan-like syndrome with loose anagen hair, cardio-facio-cutaneous (CFC) syndrome, Costello syndrome, neurofibromatosis type 1 (NF1), and Legius syndrome. This family of disorders is known as RASopathies and is one of the largest groups of multiple congenital anomaly disorders. In addition to its role in developmental disorders such as NS and RASopathies, aberrant RAS/MAPK signalling has been implicated in a wide range of leukaemias and solid tumours.

## Novel therapeutic strategies

**Fig. 1:**
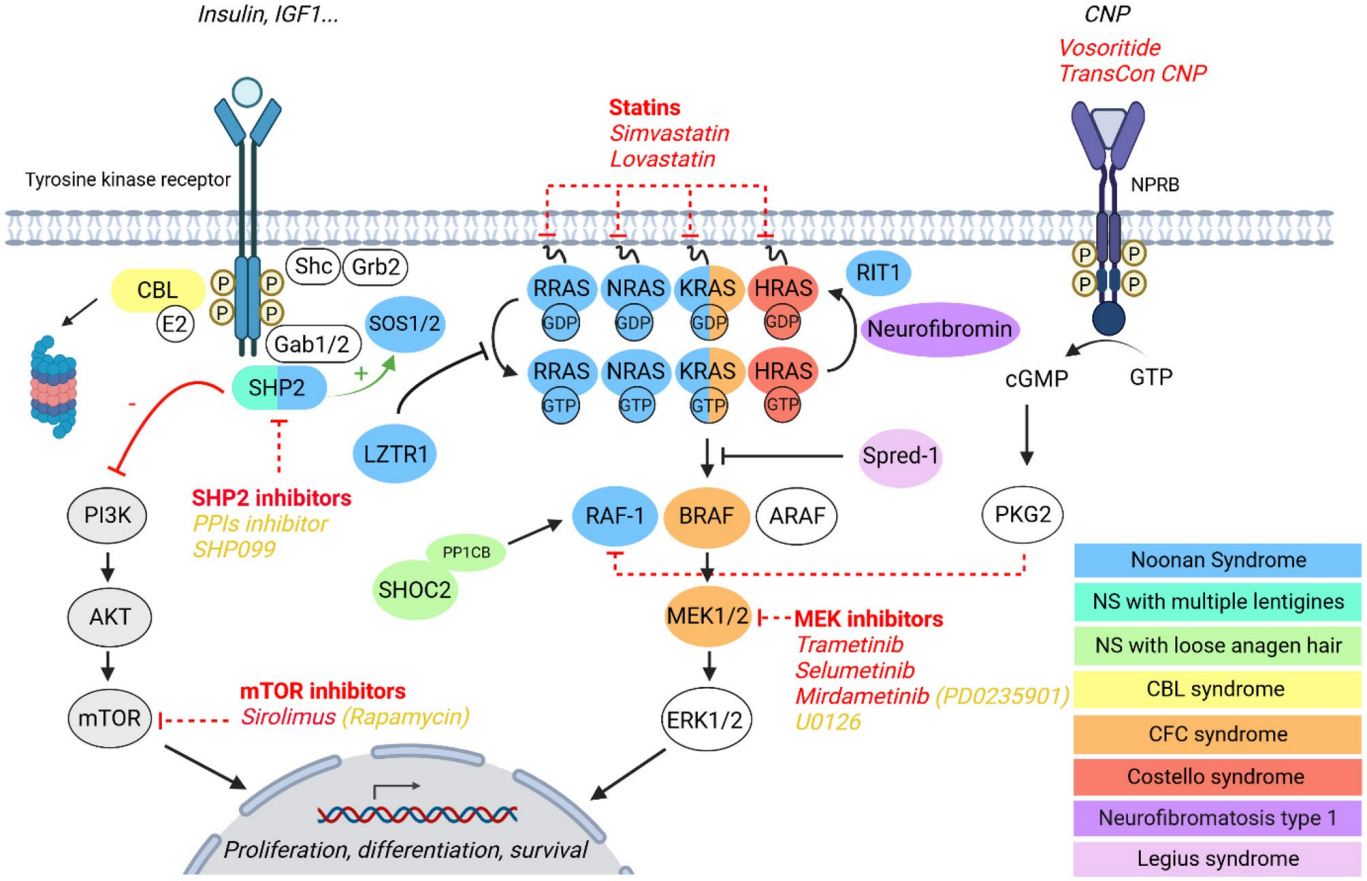
Novel therapeutic strategies in NS and RASopathies. Simplified scheme depicting the RAS/MAPK signalling pathway and disorders involving germline gain-of-function pathogenic variants of related genes as well as novel therapeutic strategies. Yellow font indicates preclinical tool compounds; white font indicates a drug in clinical development. PPIs inhibitor, protein–protein interactions inhibitor. Figure created with BioRender.com

Since the first description of NS 50 years ago, treatment of the various defects has been purely symptomatic (e.g. surgery for cardiac or skeletal defects, growth hormone treatment for short stature, or functional rehabilitation for intellectual disability). The discovery of the genetic bases of NS and other RASopathies over the last 20 years and the subsequent development of animal models has led to a better understanding of the molecular mechanisms underlying the different manifestations and helped to identify molecular targets for specific pharmacological interventions. Such treatment could correct the progressive postnatal defects of NS, such as hypertrophic cardiomyopathy, growth retardation, and cognitive impairment. Given the key role of RAS/MAPK dysregulation in the pathophysiology of NS and other RASopathies, therapeutic strategies aimed at reducing this activation appear very promising. Many specific agents (e.g. SHP2 and MEK inhibitors) have been developed and are now in clinical use for the treatment of RAS/MAPK-driven malignancies and may represent options for the treatment of patients with RASopathies. In addition, other molecules with the property to modulate RAS/MAPK activation are indicated in non-malignant diseases (e.g. C-type natriuretic peptide analogues in achondroplasia or statins in hypercholesterolemia). Drug repositioning of these molecules represents a challenging approach to treat or prevent medical complications associated with RASopathies. In this review, we will focus on therapies that have already been tested in animal models or in individuals with NS and RASopathies (Table 1). The different therapeutic strategies are illustrated in Fig. 1.

**Table 1:** Drugs already used in clinical practice in individuals with NS and RASopathies. The compounds tested in animal models are described in the text of the article

Drug	Mechanism of action	Approved drug indication	Compassionate access or clinical trials in individuals with NS and RASopathies	References
**Trametinib**	MEK inhibitor	*Melanoma, non-small cell lung cancer*	Positive effect in 3 NS infants (*RIT1*) with severe hypertrophic cardiomyopathy with heart failure	[24, 25]
			Positive effect in 6 NS children (*PTPN11*, *SOS1* and *RIT1*) and severe lymphatic abnormalities	[27, 29–31]
			No effect in one NS infant (*RAF1*) with pulmonary hypertension	[26]
**Selumetinib**	MEK inhibitor	*NF1-associated inoperable plexiform*		[33]
**Mirdametinib** (PD-0325901)	MEK inhibitor		“A phase 2b trial in adults and children with NF1-associated inoperable plexiform neurofibromas that are causing significant morbidity”	NCT03962543
**Vosoritide** (BMN111)	CNP analogue	*Achondroplasia*	“Vosoritide for selected genetic causes of short stature including NS and RASopathies”	NCT04219007
**Simvastatin**	HMG-CoA reductase inhibitor (statins)	*Hypercholesterolemia*	No effect on cognitive deficits or behavioural problems in 43 NS children (compared to 41 children with placebo)	[46]
			“Treatment with HMG-COA reductase inhibitor of growth and bone abnormalities in children with NS”	NCT02713945
**Lovastatin**	HMG-CoA reductase inhibitor (statins)	*Hypercholesterolemia*	Positive effect on synaptic plasticity as well as attention and memory in NF1 patients	[44, 45]
			“Improvement of synaptic plasticity and cognitive function in NS and NF1”	NCT03504501
**Sirolimus** (rapamycin)	mTOR inhibitor	*Prevention of organ transplant rejection*, *lymphangioleiomyomatosis*	Stabilisation of rapidly progressive hypertrophic cardiomyopathy in an NSML	[54]

### Anticancer drugs

#### SHP2 inhibitors

Given the frequency of *PTPN11* gain-of-function mutations in NS (50% of patients), SHP2 inhibitors could be a promising strategy for treating patients with this genotype. Furthermore, as SHP2 is required for full and sustained activation of the RAS/MAPK pathway in a physiological manner, its inhibition could also be of interest to reduce the pathological hyperactivation of this pathway in NS patients with other genotypes, especially if the causative mutations affect upstream components of this pathway.

SHP2 is composed of two SH2 domains at the N-terminal level (N-SH2 and C-SH2), followed by the catalytic PTP domain and a C-terminal tail with regulatory properties [6]. In the basal state, the N-SH2 domain blocks the active site of the PTP domain, thereby maintaining the phosphatase in a closed, autoinhibited conformation. Most NS-causing *PTPN11* mutations cluster at the N-SH2/PTP interface, destabilising the interaction between these two domains and causing constitutive activation of the phosphatase. Somatically acquired gain-of-function mutations in *PTPN11* are also found in sporadic malignancies such as juvenile myelomonocytic leukaemia (JMML), myelodysplastic syndromes, and acute myeloid leukaemia [7].

In recent years, several SHP2 inhibitors have been developed in the field of oncology, in particular allosteric inhibitors that maintain SHP2 in its autoinhibited conformation [8]. Because some NS-associated *PTPN11* mutations disrupt the autoinhibited conformation, the corresponding variants may be less sensitive, or even resistant, to these compounds [9, 10]. Nevertheless, the global reduction of SHP2 activity may still represent a therapeutic interest. Supporting this view, recent ex vivo experiments on blood from NS-*PTPN11* patients have shown that SHP099 can improve platelet function [11]. Recently, a new type of SHP2 inhibitor has been developed that targets the SHP2 protein–protein interactions driven by the N-SH2 domain [12]. These peptide-based molecules had nanomolar affinity for the N-SH2 domain of SHP2, good selectivity, stability to degradation, and an affinity for pathogenic variants of SHP2 up to 20-fold higher than for the wild-type protein. Interestingly, these inhibitors dose-dependently reversed the developmental defects and mortality induced by a pathogenic NS variant in zebrafish embryos (*Ptpn11*^*D61G/*+^) [12]. Furthermore, by reducing RAS/MAPK activation, SHP2 inhibitors would be expected to be effective for any genotype that causes hyperactivation of the RAS/MAPK pathway.

#### MEK inhibitors

Somatically acquired, gain-of-function mutations in *RAS* (*KRAS*, *NRAS*, and *HRAS*), leading to alterations in the RAS/MAPK pathway, are the most common cause of human cancer. As a result, many therapeutic strategies have been developed to target this pathway and may represent options for the treatment of patients with RASopathies [13, 14]. Among these strategies, MEK inhibitors have been the most extensively studied and several of them (e.g. cobimetinib, selumetinib, trametinib) are now approved for oncological indications.

Several studies have reported that treatment of NS mice with various MEK inhibitors ameliorates craniofacial abnormalities (*Ptpn11*^*Q79R/*+^ mice treated with U0126 and *KRas*^*V14I/*+^ mice treated with PD0325901) [15, 16], hypertrophic cardiomyopathy (*KRas*^*V14I/*+^, *Raf1*^*L613V/*+^, and *Sos1*^*E846K/*+^ treated with PD0325901/mirdametinib and *Rit1*^*M90I/*+^ treated with trametinib) [16–19], growth retardation (*KRas*^*V14I/*+^ and *Ptpn11*^*D61G/*+^ treated with U0126) [5, 16, 20], cognitive deficits (*Ptpn11*^*D61G/*+^ treated with SL327) [21], and lymphatic abnormalities (endothelial-specific *Raf1*^*S259A/*+^ embryos treated with U0126) [22]. It is important to note that MEK inhibitors appear to be ineffective in certain NS genotypes, such as *LZTR1* (vascular-specific *Lztr1*^*−/−*^ treated with pimasertib) [23]. In addition, for a given genotype, MEK inhibitors can rescue some manifestations but not all. For example, MEK inhibitors ameliorate growth defect and lean phenotype in NS-associated *PTPN11* mouse model but not the insulin resistance, which is driven by a direct effect of hyperactive SHP2 on macrophages (*Ptpn11*^*D61G/*+^ treated with U0126) [3]. Similarly, MEK inhibition, although effective in preventing hypertrophic cardiomyopathy, did not correct the myeloproliferative disorders of the *KRAS* mouse model [16].

Following these results in animal models, MEK inhibitors have been used as a compassionate treatment in children with severe manifestations of RASopathies. A total of three infants with *RIT1* pathogenic variants and severe hypertrophic cardiomyopathy with heart failure were treated with trametinib and showed rapid and sustained improvement in cardiac status [24, 25]. Trametinib treatment was also initiated in a premature infant with *RAF1* pathogenic variants who developed progressive hypertrophic cardiomyopathy and pulmonary hypertension [26]. Although the treatment improved the hypertrophic cardiomyopathy, the infant unfortunately died of pulmonary hypertension suggesting that MEK inhibitors are not sufficient to reverse pulmonary vascular disease. Regarding cardiovascular issues, two NS patients with *SOS1* and *RAF1* pathogenic variants with intractable multifocal atrial arrhythmia received trametinib with significant improvement within 48 h [27, 28]. MEK inhibitors may also be effective in lymphatic disorders associated with NS. Thus, a total of 6 children with NS of different genotypes (*PTPN11*, *SOS1*, and *RIT1*) and refractory chylous effusions due to lymphatic dysplasia were treated with trametinib and showed clinically significant improvement or resolution of lymphatic disease [27, 29–31]. Trametinib also efficiently reduced JMML features in a preclinical model, notably in combination with 5-azacitidine, with encouraging translational potential in patients with this disease [32].

Regarding other RASopathies, the MEK inhibitor selumetinib was approved by the Food and Drug Administration (FDA) in 2020 for the treatment of children with NF1 who have symptomatic, inoperable plexiform neurofibromas [33]. Another MEK inhibitor (mirdametinib) is currently being evaluated in NF1 patients (NCT03962543).

#### Other RAS/MAPK inhibitors

In addition to SHP2 and MEK inhibitors, many other drugs have been developed in the field of oncology for the treatment of cancers driven by alterations in the RAS/MAPK pathway and could potentially be used for the treatment of RASopathies. A comprehensive review of all compounds in development in the field of oncology can be found in recent publications [13, 14]. These compounds act at different levels of the RAS/MAPK pathway, including inhibition of tyrosine kinase receptor kinase activity (e.g. dasatinib), inhibition of RAS activity (e.g. sotorasib), RAS membrane localisation (e.g. tipifarnib and lonafarnib) or RAS interaction with its exchange factor SOS1 (e.g. BI-1701963 and RMC-5845), inhibition of RAF (e.g. belvarafenib), or MEK (e.g. ulixertinib and LY3214496). These agents have not yet been tested in animal models or individuals with NS or RASopathies, but may represent a therapeutic option in the future.

### C-type natriuretic peptide analogues

The C-type natriuretic peptide (CNP) signalling pathway consists of CNP, its receptor, the natriuretic peptide receptor-B (NPR-B), and its effector, the cGMP-activated kinase 2 (PKG2). Activation of this pathway leads to the inhibition of RAF-1, thereby reducing RAS/MAPK activation [34, 35]. Although CNP is mainly produced in the growth plate, it is also produced by a variety of tissues, including the brain and vascular endothelial cells [36]. It functions as an autocrine/paracrine regulator, particularly in the brain, the growth plates, and cardiovascular tissues, which opens up interesting perspectives for the treatment of RASopathies [37]

In recent years, CNP analogues have been developed for the treatment of achondroplasia, one of the most common skeletal dysplasias with pathophysiological similarities to RASopathies. Indeed, achondroplasia is caused by a gain-of-function mutation in the fibroblast growth factor receptor 3 (*FGFR3*) gene, a member of the tyrosine kinase family, resulting in prolonged activation of RAS/MAPK and alteration of chondrocyte proliferation and differentiation at the growth plate level. Although more moderate, similar growth plate abnormalities have been described in a mouse model of NS (*Ptpn11*^*D61G/*+^) [37] and also in a mouse model of CFC syndrome (*Braf *^*Q241R/*+^) [38]. An analogue of CNP that is resistant to proteolytic degradation (BMN111/vosoritide; BioMarin) was generated in 2006. Treatment with BMN111 in a mouse model of achondroplasia significantly improved growth plate abnormalities and bone growth by reducing RAS/MAPK activation [39]. Subsequently, results from a phase 2 dose-finding and extension study (NCT02055157 and NCT02724228) of vosoritide in children with achondroplasia demonstrated a sustained increase in annualised growth velocity for up to 4 years [40, 41]. Based on these results, vosoritide has recently been approved for the treatment of children with achondroplasia. Another CNP analogue conjugated to a polyethylene glycol carrier molecule via a cleavable linker, which allows for a longer half-life (TransCon CNP; Ascendis), is currently being evaluated in a phase 2 clinical trial (NCT04085523).

Interestingly, treatment with CNP has been reported to increase body length in a mouse model of CFC syndrome (*Braf *^*Q241R/*+^) [38]. A clinical trial of vosoritide for the treatment of short stature in children with NS and RASopathies is ongoing (NCT04219007). Three children with *PTPN11* pathogenic variants included in this trial, who have completed 12 months of vosoritide treatment, show improvement in growth velocity.

### Statins

Statins are 3-hydroxy-3-methylglutaryl coenzyme A (HMG-CoA) reductase inhibitors widely used in the treatment of hypercholesterolaemia in children and adults. By negatively regulating the mevalonate pathway, statins inhibit the synthesis of cholesterol, but also the synthesis of substrates required for prenylation, a necessary step in the localisation and activation of RAS at the cellular membrane level. As a result, this treatment has been suggested as a potential therapy for RASopathies.

Statins were first investigated in the treatment of cognitive impairment in NF1. Two studies have reported that treatment of NF1 mice (*Nf1*^±^) with lovastatin improved both synaptic plasticity and cognitive function [42, 43]. Subsequently, lovastatin was shown to improve synaptic plasticity as well as attention and memory in NF1 patients [44, 45]. This positive effect of statins on cognition was not reproduced with simvastatin [46].

The efficacy of statins (lovastatin and rosuvastatin) has also been reported in NS mice (*Ptpn11*^*D61G/*+^) for the treatment of learning and memory deficits [21] as well as growth plate abnormalities [37]. Statins (simvastatin, atorvastatin, and fluvastatin) also improved survival in *Drosophila* NS model [47]. Based on these findings, two clinical trials of statins in RASopathies are currently underway: one testing simvastatin for the treatment of growth and bone abnormalities in children with NS (NCT02713945) and the other testing lovastatin for the improvement of synaptic plasticity and cognitive function in children with NS or NF1 (NCT 03504501).

### Pathophysiology-driven approaches

A better understanding of the pathophysiology of NS and other RASopathies has also highlighted specific defects at the subcellular, cellular, or tissue levels that may represent potent targets for selective therapies. Thus, recent preclinical studies in mouse and zebrafish models of NS have shown constitutive inflammation as a driving force for metabolic impairment and haematological features [3], and treatment of zebrafish embryos with the anti-inflammatory corticosteroid dexamethasone improved the JMML phenotype [48]. In addition, dysfunctions of mitochondrial bioenergetics and quality control have been identified in several RASopathies, including NS and Costello syndrome, that are causally linked to heart disease [49, 50]. Importantly, Dard and colleagues [49] recently described that pharmacological rescue of mitochondrial function improved hypertrophic cardiomyopathy in mouse and zebrafish models of Costello syndrome.

## Conclusion and perspectives: challenges in the treatment of RASopathies

The identification of novel specific therapies opens up very promising and exciting perspectives in the management of patients with RASopathies. However, many issues and pitfalls remain to be resolved.

## Which patients to treat and why?

The main potential therapeutic targets for the RASopathies include progressive postnatal defects, such as hypertrophic cardiomyopathy, growth retardation, and cognitive deficit. To date, most of the patients who were treated with new therapies, such as MEK inhibitors, presented severe manifestations of RASopathies for which no conventional therapy is available (e.g. severe hypertrophic cardiomyopathy and lymphovascular disease). However, these severe manifestations affect only a limited number of individuals, making it difficult to conduct clinical trials. New therapeutic strategies aimed at preventing more common and less severe manifestations (e.g. short stature and cognitive deficits) have the potential to benefit large numbers of people, but may also raise safety issues. In addition, symptomatic treatments are already available for some of these defects, such as growth hormone therapy for short stature. Of course, the interest of a more pathophysiological treatment would be to improve several manifestations simultaneously. Ongoing clinical trials (e.g. statins and CNP analogue) are likely to help us to move forward on this issue.

## Which agent to use, at what dosage?

Data obtained from animal models of NS suggest that the different therapeutic strategies may be effective in some genotypes, but not all. For example, allosteric inhibitors of SHP2 are not effective in the NS-associated *PTPN11* genotype [9], nor are MEK inhibitors in the *LZTR1* genotype [23]. Furthermore, one therapy may not be sufficient to correct all the manifestations associated with a given genotype. For example, NSML-associated *PTPN11* mutants promote PI3K/AKT/mTOR hyperactivation and a mTOR inhibitor (sirolimus) improved NSML-associated hypertrophic cardiomyopathy in animal models and patients [51–54]. Combination therapy, or the sequential use of different therapies, may be a possible therapeutic strategy. It is important to note that in the field of oncology, MEK inhibitors are usually combined with other anticancer therapies. Indeed, due to the disruption of a negative feedback loop to the RAF proteins, MEK inhibitors used as monotherapy show only modest efficacy [32, 55].

The choice of drug dose is also important to limit potential side effects. Interestingly, the clinical efficacy of MEK inhibitors, for example, is observed at lower doses than the recommended cancer dose [24]. This may be explained by the fact that RAS/MAPK hyperactivation is lower in developmental disorders such as RASopathies than in acquired cancers.

## When to treat and for how long?

An important issue is the definition of the optimal age for starting treatment. In several animal studies, MEK inhibitor treatment was initiated during embryonic development (by exposing the pregnant mothers to the MEK inhibitor) and continued after birth [15, 16, 23]. In these studies, the effect of the MEK inhibitor may vary depending on the timing of the treatment. For example, in a *KRas* mouse model, MEK inhibition prevented the various developmental defects (i.e. craniofacial, cardiac, and growth defects) when started prenatally, whereas this treatment did not ameliorate these defects when started after weaning, highlighting the importance of the timing [16].

Another issue is the duration of treatment, which needs to be longer for RASopathies than for cancer. Therapy aimed at allowing normal growth and cognitive development should probably be maintained throughout childhood. In this case, treatments with fewer side effects than anticancer drugs (e.g. statins or CNP analogues) could be proposed.

## How to assess the efficacy and safety of these treatments?

The ability to include individuals with RASopathies in therapeutic trials is a real challenge due to the rarity of the disease, the phenotypic variability between patients (even within the same family), and the lack of knowledge about the natural history of the disease. This is even more true when the different genotypes are taken into account. Only international clinical trials will be able to answer these questions.
